# skillsChain: A Decentralized Application That Uses Educational Robotics and Blockchain to Disrupt the Educational Process

**DOI:** 10.3390/s21186227

**Published:** 2021-09-16

**Authors:** Panayiotis Christodoulou, Andreas S. Andreou, Zinon Zinonos

**Affiliations:** 1 Intelligent Systems Laboratory, Department of Computer Science, Neapolis University Pafos, Pafos 8042, Cyprus; panayiotis.christodoulou@nup.ac.cy; 2 Department of Electrical Engineering, Computer Engineering and Informatics, Cyprus University of Technology, Limassol 3036, Cyprus; andreas.andreou@cut.ac.cy

**Keywords:** educational robotics, Blockchain, decentralized application

## Abstract

Our epoch is continuously disrupted by the rapid technological advances in various scientific domains that aim to drive forward the Fourth Industrial Revolution. This disruption resulted in the introduction of fields that present advanced ways to train students as well as ways to secure the exchange of data and guarantee the integrity of those data. In this paper, a decentralized application (dApp), namely skillsChain, is introduced that utilizes Blockchain in educational robotics to securely track the development of students’ skills so as to be transferable beyond the confines of the academic world. This work outlines a state-of-the-art architecture in which educational robotics can directly execute transactions on a public ledger when certain requirements are met without the need of educators. In addition, it allows students to safely exchange their skills’ records with third parties. The proposed application was designed and deployed on a public distributed ledger and the final results present its efficacy.

## 1. Introduction

Currently, the rapid technological developments in the areas of computing and robotics lead into the introduction of new emerging fields. Two growing fields that have the prospect of meaningfully impacting science and technology education are the fields of educational robotics [1] and Blockchain [2]. The aim of educational robotics is to enhance skills development by exploiting robots, while Blockchain’s purpose is to offer secure and transparent ways to record and transfer data.

To understand deeply how Blockchain can actually aid education and more specifically the area of educational robotics, it is important to recognize its unique characteristics [3,4] as any one or more may be adapted for educational uses. Firstly, Blockchain provides traceability; as every block is linked together with the previous one, a user can track the executed transactions conducted on a public ledger with ease. Secondly, Blockchain guarantees trust and removes the need for intermediates as the verification of data in a Blockchain network is performed by the participants of the overall p2p network and not from a central party. Thirdly, Blockchain ensures security as its braces various security mechanisms such as consensus algorithms, hashing, and encryption to overhaul the network’s conditions and data. Lastly, Blockchain offers decentralization; due to its architecture, it provides the capacity to operate numerous transactions between accounts without maintaining expensive centralized data centers.

Nowadays, there has been a growth demand on the implementation of decentralized applications (dApps) in various domains [5,6,7] as an attempt to exploit the numerous capabilities of Blockchain technology; however, despite this highly increased demand, educational robotics is an area that lacks from dApps’ development.

This work introduces skillsChain, a fully deployed Ethereum dApp developed using Solidity that exploits the capabilities of Blockchain to provide means to educational robotics for securely recording students’ skills during the educational process. The aim of the proposed dApp is to provide an approach for securely exchanging students’ records starting from their early years in education and beyond. As students’ skills are continuously developed by a series of tasks/activities that students undertake while in schools, the recording of students’ skills will be also beneficial beyond the borders of academia [8]. The following research questions motivated this paper:

1. How can Blockchain technology aid in educational robotics?
2. How can educational robotics securely record students’ skills during the educational process?
3. How can students exchange and use their data in higher education and beyond?

In sequence, to present thorough answers to the above-noted questions, a decentralized smart contract was developed and executed on Ethereum [9]. As identified in the literature, educational robotics is an area in which the use of distributed ledger technologies such as Blockchain is not present. In this work, we outline a forward-looking architecture in which ER can directly execute transactions on a public ledger when certain requirements are met without the need of educators. The framework as well as the results of the proposed approach are discussed in the following sections.

## 2. Literature Review

This section provides an overview on educational robotics and Blockchain-based smart contracts and then focuses on the current progress of Blockchain within education.

### 2.1. Educational Robotics

In the last years, an increased demand in robotics has been noted, refining various domains of applications such as path planning [10], picking, and packing. By taking into account the effect of robots in numerous scientific fields, nowadays, robotics have also been introduced in education. Educational robotics (ER) is a newly presented topic of research that intends to enhance the educational experience through the formation, implementation, and validation of pedagogical tasks [11,12]. The authors in [13] outline the significant importance of ER for educational communities and specify the ER term according to the core fields involved, namely education, robotics, and HCI (human–computer interaction). In [14], authors present the growth in assisted education using special-purposed social interaction games. More specifically, in [14], the authors present robot games in which kids with autism can interact with robots to evaluate the advantages of a more interactive therapy. Experimental results strongly suggest that robots offer various benefits to children with autism.

As stated in the literature, there are numerous processes for which ER can be utilized in the educational procedures (i) as a tool to learn or teach; (ii) as a generic subject with its own curriculum; and lastly (iii) as a tool which could aid students in enhancing their skills. Taking the last statement into consideration, the study in [15] defines a list of skills that can be developed and improved by using educational robotics. This list includes but is not limited to adaptive and critical thinking skills, collaboration and communication skills, problem solving skills, creativity skills, and finally management skills. The above-mentioned skills were taken into consideration when designing skillsChain.

### 2.2. Blockchain and Smart Contracts

Bitcoin, the world’s premier Blockchain application, was initially introduced as a solution to tackle the double-spending problem [16] in order to become the world’s first digital currency not vulnerable to attacks. Bitcoin presented a newly developed consensus algorithm, namely proof-of-work, as a mean to verify the transactions that take place in a peer-to-peer (p2p) network to provide trusted and transparent transactions. Bitcoin conferred to researchers as well as to software developers the opportunity to learn, explore, and expand the use of Blockchain technology to what we observe nowadays in our daily lives. Currently there are various Blockchain frameworks that offeri decentralized solutions in numerous fields of applications and one of those frameworks is the Ethereum network. The Ethereum Blockchain network was introduced several years after the launch of Bitcoin as a framework that empowers the creation of smart contracts for the next-generation of web apps, namely decentralized applications (dApps). The definition of smart contracts is not something new to the scientific world as they were firstly introduced by Nick Szabo back in 1997 [17], but their usage never took place due to the fact that there was not a proper infrastructure that could support the use of smart contracts up until the introduction of Blockchain technology. A smart contract is defined as a self-executable code with certain rules that can be deployed into a Blockchain network. Those rules are automatically triggered on a Blockchain network when specific conditions are met. In the Ethereum network, smart contracts are activated by transactions that are transmitted to the Blockchain nodes through function invocations. Smart contracts allow trusted transactions to take place between various users, eliminating the need of central authorities. In addition, smart contract transactions are recorded on a public ledger that is impossible to modify as it inherited the capabilities of Blockchain technology.

Although, the Ethereum network was the first publically distributed ledger that enabled various parties to develop secure dApps, nowadays, the interest of the scientific community extends from the design and development of special-purposed smart contracts to solutions that combine off-chain and on-chain data using external APIs such as Chainlink (https://chain.link/ (accessed on 10 September 2021)), as well as to approaches that could outsource specific tasks on cloud-servers and then use a Blockchain network to verify the computational outcomes, such as the work presented in [18].

### 2.3. Blockchain in Education

Even though currently there is an increased demand for the development of dApps in various domains, the field of education robotics lacks recognition of the development from dApps, thus, in this sub-section, we first concentrate on the benefits that Blockchain can offer in education and then we present current works that make use of Blockchain in education.

According to [19], Blockchain technology has endless opportunities to become a comprehensive part of the educational system. The adoption of Blockchain into the educational field can offer decentralization, reliability, security, as well as reductions on administrative costs. Despite the possibilities that Blockchain can offer in the educational domain, authors in [19] identify limitations on compliance, data ownership, and authenticity of data sources that should also be taken into account when designing a decentralized application. In this context, authors in [20] present a personal data broker that acts as an additional privacy layer for smart contracts, which could be used for data privacy assurance to prevent leaks or the misuse of data about the students or their activity.

The last few years, Blockchain introduced applications that can be utilized within the educational world to make teaching and learning more engaging [21], enhance the educational process, and motivate lifelong learning. In this context, the authors in [22] present the Blockchain for Education platform, which is used for issuing, validating, and sharing purposes. The proposed platform is implemented on the Ethereum network and uses the Interplanetary Filesystem (IPFS) for storing purposes. Experimental evaluation presented that the developed prototype enables tamper-proof archiving of certificates, the correct distribution of certificates to learners, as well as a verification mechanism for certificate validation. In addition, the work in [23] proposes a similar solution. Authors in [23] present a novel Blockchain-based approach in which students can be the curators of their educational records, allowing them at the same time to share their records with other trusted parties. By utilizing the proposed solution, employers can verify candidates’ educational records and observe what the candidate has done related to the job he/she is applying for. Unlike the work in [22], authors in [23] have implemented their own private Blockchain framework to manage educational records, thus the main architecture includes various nodes such as the provider, the individuals, and the miner nodes. The evaluation of the proposed architecture exposed that it can provide education a new way for incorporating sensitive data with success.

In addition, the authors in [24] explore the Blockchain technology to observe if the OpenSource framework behind cryptocurrencies can provide any improvements to the existing online learning experience. Devine [24] presents two use cases to support its study. In the first case, Devine presents the ability of students to move between institutions, simultaneously keeping an open verifiable record of their learning achievements. In the second example, Devine proposes a solution in which institutions can use the Blockchain network to offset the costs of learning. In parallel, another influential example in the learning engagement area is ODEM [25]. ODEM.io aims to revolutionize how educators and learners plan, connect, and book educational programs. ODEM uses its own token economy to reward educators who create and teach programs, while learners use those tokens to enroll in a specific program tailored to their interests.

In addition, many other private initiatives focus on making the learning process more effective. For instance, BitDegree [21] offers a gamified online education platform that provides incentives to learners when completing tech courses. In this context, BitDegree aids educators in building Blockchain-based gamified experiences, which are then offered to learners.

As identified in the literature, direct links between educators and learners, as well as gamified experiences, play a critical role for the mass adoption of Blockchain technology in education. The proposed work presents a state-of-the-art approach that combines Blockchain and educational robotics to securely record the development of students’ skills, simultaneously removing any intermediate actions needed to be taken from educators.

## 3. skillsChain Architecture

**Figure 1:**
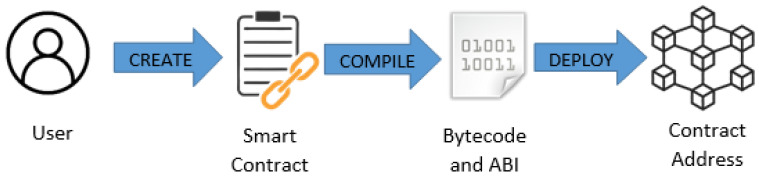
Contract compilation and deployment.

The proposed methodology utilizes Blockchain technology through the functionality of a special-structured smart contract that was created to evaluate its use. The smart contract ensures encryption and hence secure transfers of information. The purpose of skillsChain is three-fold: (i) permit the contract owner (educator) to grant access to authorized users (educational robots); (ii) allow authorized users (educational robots) to create and update student records after the completion of a task/activity; and finally (iii) allow students to view, read, and share their skills’ records. The proposed smart contract was firstly developed on Solidity [25] and then was deployed on the Rinkeby Network to evaluate its effectiveness under real-world conditions. We note that the smart contract can be executed on any Blockchain network that runs the Ethereum Virtual Machine (EVM). Figure 1 outlines the flow of the contract compilation and deployment on EVMs. At first, the contract owner creates the smart contract based on a set of specific requirements. Subsequently, the smart contract is compiled and its Bytecode, along with its Application Binary Interface (ABI), are created (the Bytecode as well as the ABI consist of essential parts for the deployment of the contract). At the final stage, the contract is deployed to the Ethereum network and a contract address that refers to the specific smart contract is generated. The users of the smart contract can execute, write, or read functions by using the specific contract address.

**Figure 2:**
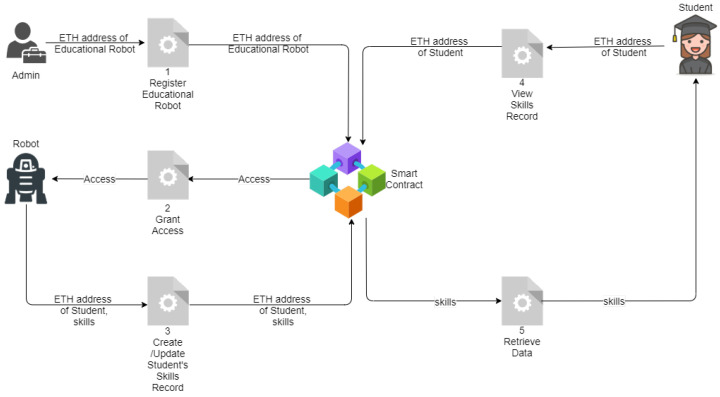
A schematic representation that presents the proposed workflow.

The proposed dApp, namely skillsChain, consists of three main actor: the contract owner, as known as the administrator; the authorized users, as known as the educational robots; and finally the students. The workflow of the proposed mechanism is depicted in Figure 2 and involves a series of steps which are outlined below.

1. Firstly, the administrator of the smart contract registers an educational robot on the Blockchain by providing the robot’s Ethereum address.
2. When the transaction is successfully executed on the Blockchain, the specific educational robot grants access on the smart contract of the proposed dApp, thus it can create or update records.
3. At stage 3, educational robots observe a student when he/she undertakes a predefined task/activity that is supported by the robot. The tasks/activities may include but are not limited to speaking, mathematics, memory or mime games, etc. The aim of the proposed work is not to deeply examine the tasks/activities offered by educational robots [15,26] or which skills are developed when students undertake specific tasks/activities, but rather to propose a decentralized solution for educational robots that can automatically create or update a student record at the completion of a task/activity. In this context, at stage 3, when a certain task/activity concludes, the educational robot creates a new or updates the existing record of the student using the student’s Ethereum address as well as the values of certain skills, such as: adaptive and critical thinking skills, collaboration and communication skills, problem solving skills, creativity skills, and finally management skills. We note that students’ skills are directly sent on the Blockchain without being inferred by the educator.
4. When the transaction of the educational robot is successfully verified on the Blockchain network, the skills of the specific student are recorded on the smart contract; thus, the student, by using his/her unique Ethereum address, can interact with the smart contract to retrieve its data.
5. At any time, students can share their Ethereum address with other parties who can also interact with the smart contract to view the developed skills of the student.

**Figure 3:**
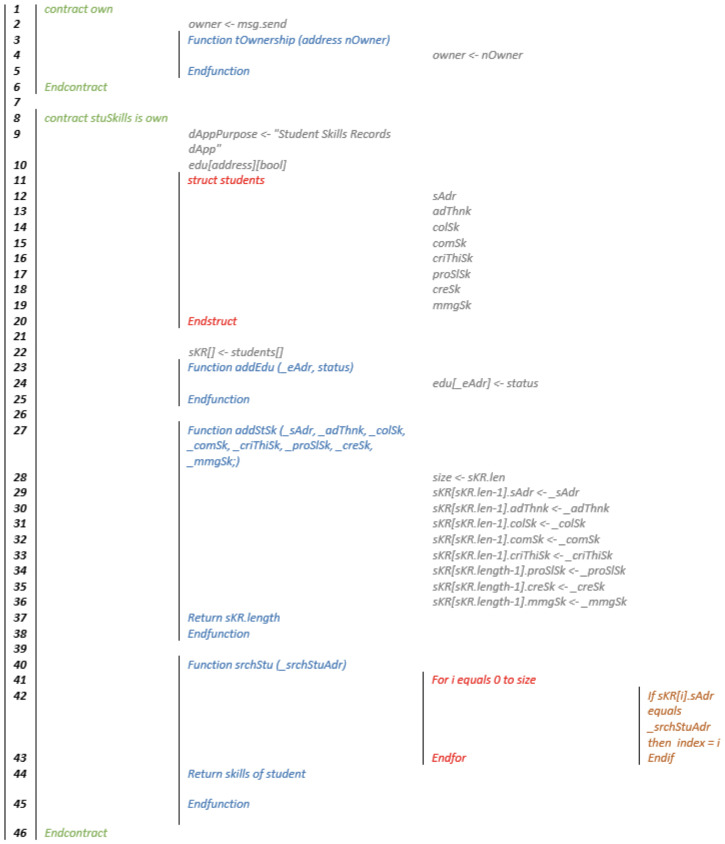
Pseudo code of skillsChain dApp.

The reader can refer to the following GitHub repository for complete access to the source code of the smart contract (source code: https://github.com/panayiotis-christodoulou/skillsChain (accessed on 10 September 2021)). In addition, Figure 3 presents the pseudocode of the proposed smart contract.

## 4. Use Case Scenario

A use case scenario was setup to evaluate the efficiency of skillsChain. Firstly, we note that skillsChain’s smart contract was deployed on the Rinkeby Test Network and the list of executed transactions can be found on https://rinkeby.etherscan.io/address/0xDD311513CDa396A8ba53ddaF08eeef27De5a489B (accessed on 10 September 2021). In the following scenario, the contract owner, with an ETH address (0xea5EE8c830b4881c4e5315B572fe5973b0e3e1E0), acts as the administrator of skillsChain, thus it can register robots which act as educators.

To evaluate the effectiveness of the addEducator(…) function, we firstly generated a new ETH address for a specific robot (0x91b8Ee9d8FFad82d9E00ba3e4cB7C4a7D44B5A67) and then executed the addEducator(…) function on the Rinkeby Test Network using the robot’s ETH address and a status value. The purpose of the educator status attribute (bool variable) is to grant or remove access; a value of 1 means that you grant access to the specific ETH address for interacting with the smart contract, while a value of 0 means that you revoke the interaction capabilities of the specific robot.

In the proposed scenario, the robot with an ETH address (0x91b8Ee9d8FFad82d9E00ba3e4cB7C4a7D44B5A67) was granted access on the smart contract; thus, when a certain task/activity completes, the robot can create or update a student’s skills record. In this context, we generated an ETH address for a specific student (0x36F4bD81feaCcAb36c1152590C58E77a6f23eb1e), which was later used by the robot to create his/her skills record. In this case, our aim was to evaluate the effectiveness of the addStudentSkills(…) function. Table 1 presents the values that were sent to the smart contract directly from the robot for that specific student when the student completed a task.

As can be observed from Table 1, the values of the same skills are low. This is due to the fact that the certain task/activity undertaken by the student was not ideal for developing that specific skills.

**Figure 4:**
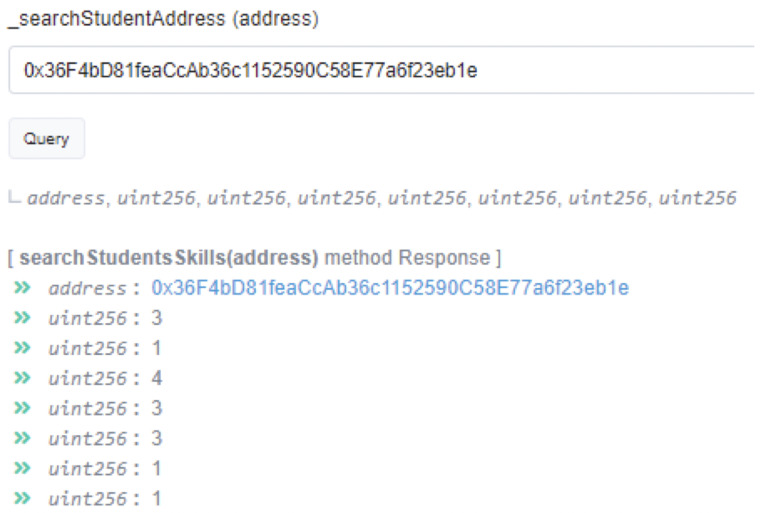
Real-time observed skill values for the specific student.

Furthermore, Figure 4 demonstrates the use of the searchStudentsSkills(…) function. When a user calls the searchStudentSkills() function, the smart contract retrieves the data for a specific student. In the proposed dApp, the searchStudentsSkills(…) function was executed by providing the ETH address of the specific student (0x36F4bD81feaCcAb36c1152590C58E77a6f23eb1e) and the student’s record was successfully retrieved. At any point, students can share their Ethereum address with a certain party, which in return can self-verify their skills.

The aforementioned scenario presented the effectiveness and usage of the proposed methodology, which firstly aimed to allow educational robotics to securely record students’ skills during the educational process and secondly to permit students to share or view their records with higher education institutions and beyond.

## 5. Discussion on Timing and Fees

In this section, a discussion on timing and fees per transaction is presented. Table 2 presents the minimum values of gas along with the fees in USD that are needed for the smart contract deployment as well as for executing the main functions of the smart contract. Fees were calculated by taking into consideration the current price of Ethereum (ETH: USD 1785). It can be observed that deploying the smart contract on Ethereum costs around USD 2, while executing the main functionalities varies from USD 0.13 to USD 0.65.

In addition, the average execution times for calling the core functions of the proposed app are outlined in Table 3. For the addEducator() function, an average time of 6 s is required before the transaction can be verified on Ethereum, while the addStudentSkills() function requires an average time of 10 s. The reading functions, such as searchStudentsSkills(), do not require any gas, thus an average time of some milliseconds is needed to retrieve the data.

In the future, to address the economic issues that may arise due to the high price of Ethereum or to tackle any scalability challenges that may come into sight, the proposed solution can be deployed on any other Blockchain framework that supports the Ethereum Virtual Machine (EVM), such as Polygon, Moonriver, and Binance Smart Chain, and offers lower transaction fees as well as better scalability mechanisms.

## 6. Conclusions

This paper presented skillsChain, a state-of-the-art decentralized application implemented on Solidity and deployed on the Ethereum network, that exploits the capabilities of Blockchain to provide means to educational robotics for securely recording students’ skills during the educational process. The aim of the proposed app was to provide a real-world solution in which educational robots can directly interact with the Blockchain without being inferred by a third party. The experimental scenario that was designed and tested under real-world conditions outlines the effectiveness of the proposed architecture. Due to the fact that in the proposed architecture, the core components are not directly linked, human effort is partially required. In the future, we aim to fully automate the proposed solution by utilizing computer vision techniques in which the verification of the student will be performed directly from the robot by firstly outsourcing the verification process and then by use Blockchain to verify the outcome before the execution of specific tasks by students.

## Figures and Tables

**Table 1:** Skills development for the certain student.

studentAddress	0x36F4bD81feaCcAb36c1152590C58E77a6f23eb1e
adaptiveThinking	3
collaborationSkills	1
communicationSkills	4
criticalThinkingSkills	3
problemSolvingSkills	1
creativitySkills	1
managementSkills	1

**Table 2:** Transaction fees on skillsChain.

Deployment/Functions	Gas Price (Gwei)	Gas Limit	Transaction Fee (Ether)	Transaction Fee (USD)
Contract deployment	1	712,671	0.0007126	1.98
addEducator()	1	46,335	0.000046	0.13
addStudentSkills()	1	229,282	0.00023	0.64

**Table 3:** Execution time per function.

Function	Average Execution Time (s)
addEducator()	6
addStudentSkills()	10
searchStudentsSkills()	<1

## Data Availability

Not applicable.
